# Advances in cryoEM and its impact on β-pore forming proteins

**DOI:** 10.1016/j.sbi.2018.07.010

**Published:** 2018-10

**Authors:** Courtney M Boyd, Doryen Bubeck

**Affiliations:** Department of Life Sciences, Imperial College London, South Kensington Campus, London SW7 2AZ, UK

## Abstract

Deployed by both hosts and pathogens, β-pore-forming proteins (β-PFPs) rupture membranes and lyse target cells. Soluble protein monomers oligomerize on the lipid bilayer where they undergo dramatic structural rearrangements, resulting in a transmembrane β-barrel pore. Advances in electron cryo-microscopy (cryoEM) sample preparation, image detection, and computational algorithms have led to a number of recent structures that reveal a molecular mechanism of pore formation in atomic detail.

## Highlights

• Advances in sample preparation of membrane proteins have improved imaging conditions for beta-PFPs.
• Hardware and software innovations in cryoEM enable computational strategies that separate pores of mixed stoichiometries.
• High-resolution cryoEM structures of β-PFPs reveal a general mechanism of pore formation conserved across diverse family members.
• CryoEM structures of beta-PFPs reveal a general mechanism of pore formation conserved across diverse family members

**Current Opinion in Structural Biology** 2018, **52**:41–49

This review comes from a themed issue on **Cryo electron microscopy**

Edited by **John Briggs** and **Werner Kuhlbrandt**

For a complete overview see the Issue and the Editorial

Available online 17th August 2018

**https://doi.org/10.1016/j.sbi.2018.07.010**

0959-440X/© 2018 The Authors. Published by Elsevier Ltd. This is an open access article under the CC BY license (http://creativecommons.org/licenses/by/4.0/).

## Introduction

The outer membrane of a cell provides an essential barrier from its external surroundings and creates a unique chemical environment for cellular processes. Pore-forming proteins (PFPs) puncture cell membranes allowing passage of solvent and often proteins into the target cell. Pore-forming proteins are exploited by a wide range of organisms as a mechanism to lyse target cells. Based on the secondary structure of their transmembrane regions, PFPs are classified into two broad subgroups (α and β). Both families undergo a transition from soluble monomeric proteins to membrane-embedded oligomeric assemblies [1]. Dramatic structural changes accompany this transition. Historically, X-ray crystallography has yielded high-resolution information for soluble states, while electron cryo-microscopy (cryoEM) provided low-resolution reconstructions of oligomeric membrane-associated complexes (Table 1). Advances in membrane-protein biochemistry, coupled with recent technical developments in cryoEM, have led to a burst of high-resolution structures of transmembrane complexes (Table 1). This review highlights the impact of cryoEM on recent β-PFP structures, focusing on the seminal work on the anthrax toxin protective antigen (PA) pore and later structures from the aerolysin and membrane attack complex perforin/cholesterol dependent cytolysin (MACPF/CDC) super-families.

**Table 1:** Single-particle cryoEM structures of β-PFPs in their transmembrane states

β-PFP	Membrane environment	Resolution	Detector	Motion-correction	3D classification	Reference
Pneumolysin	Liposome	28 Å	Film	No	No	[27]
Perforin	Liposome	28.5 Å	CCD	No	No	[29]
Anthrax	Nanodisc	22 Å	Film	No	No	[10]
Pleurotolysin	Liposome	11 Å	CCD	No	No	[26]
Suilysin	Liposome	15 Å	CCD	No	No	[30]
Anthrax	Detergent^a^	2.9 Å	DED	Yes	Yes	[12^••^]
MAC	Detergent	8.5 Å	DED	Yes	Yes	[25^••^]
Pneumolysin	Amphipol	4.5 Å	DED	Yes	Yes	[34^••^]
Lysenin	Detergent	3.1 Å	DED	Yes	Yes	[18^••^]
Aerolysin	Detergent	7.9 Å	DED	Yes	Yes	[19^••^]
Gasdermin	Detergent	3.8 Å	DED	Yes	Yes	[36^••^]

a Soluble toxin triggered to transmembrane pore on grid support, detergent subsequently added. DED, direct electron detector; CCD, charge-coupled device.

β-PFPs have been visualised by negative stain and cryo-microscopy for nearly forty years. However, difficulties in stabilizing membrane proteins outside the lipid bilayer, together with heterogeneity of oligomeric assemblies, has made it challenging to characterise these complexes at a resolution necessary to detail a molecular mechanism of pore formation. Nonetheless, the excitement around the possibilities enabled by the ‘resolution revolution’ in cryoEM have made it an opportune time to re-visit these questions.

Fundamental advances in both hardware and software developments underpin all the recent sub-nanometer pore structures. Most notably, the use of direct electron detectors (DED) in data acquisition has provided a step change in increasing the signal-to-noise ratio of experimental images [2], and their rapid read-out has enabled the recording of movie frames. All of the high-resolution β-PFP structures were collected with direct electron detectors and used computational strategies to correct for beam-induced specimen motion across image frames [3]. Empirical Bayesian-based algorithms for image processing have also been instrumental in refinement of 3D structures [4,5]. This approach has proven most powerful in the classification of mixtures within samples [6], an important consideration for structural studies of notoriously heterogeneous pore and prepore assemblies.

## Anthrax toxin protective antigen (PA) pore

Anthrax toxin is the major virulence factor for the pathogen *Bacillus anthracis*, and is a tractable model system for studying protein translocation. The toxin’s protective antigen (PA) binds to receptors on the target cell plasma membrane, oligomerizes into a heptameric prepore, and is endocytosed. Upon acidification, PA undergoes the transition to a transmembrane pore that translocates two enzymes, lethal factor and edema factor, into the cytosol of the infected cell [7].

Like many β-PFPs, anthrax toxin PA is an heterogeneous oligomeric assembly that is prone to aggregation when removed from a membrane environment [8]. To get around this, early structural studies of PA bound the toxin to chaperone GroEL and used it as a molecular scaffold when triggering the prepore-to-pore transition [9]. As model membrane systems evolved, the PA pore was reconstituted in both liposomes and lipid nanodiscs before imaging by negative stain EM [10]. Later, low-resolution cryoEM was used to visualize a domain of lethal factor bound to the PA pore inserted in a lipid nanodisc [11]. However, it was not until pores were formed directly on the carbon support of an EM grid pre-treated with polylysine that enough monodisperse complexes could be embedded in a thin layer of ice for high-resolution studies [12^••^]. An automated data-collection strategy enabled thousands of micrograph movies to be recorded on a DED. Beam-induced specimen motion was corrected across movie frames to further increase the signal-to-noise ratio in the images. Within a Bayesian framework, particles were classified at both the two-dimensional and three-dimensional levels to achieve a homogeneous subset that finally reached high-resolution.

**Figure 1:**
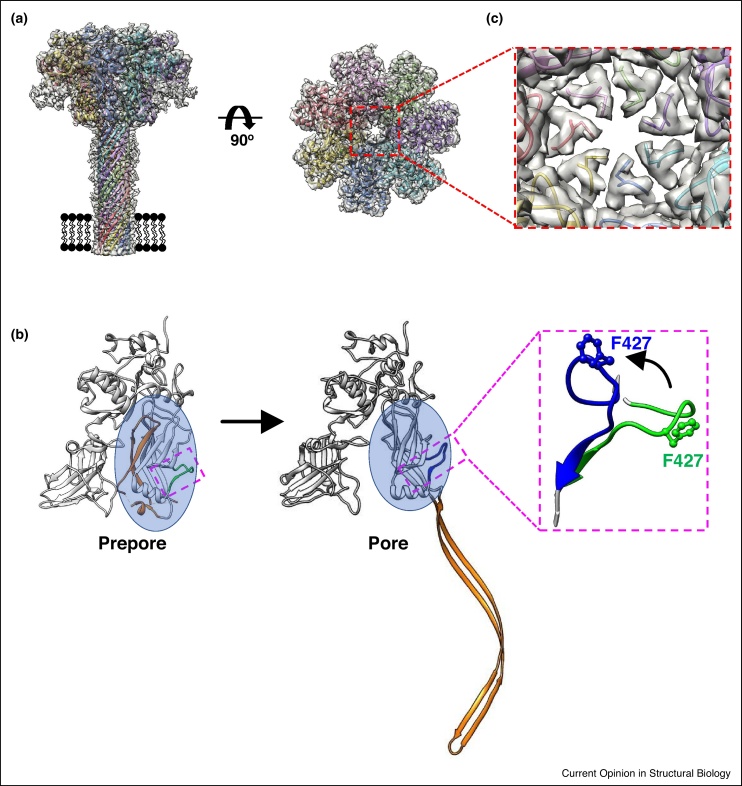
Mature anthrax toxin PA pore at 2.9 Å resolution. **(a)** CryoEM reconstruction of the heptameric PA pore structure (grey transparent surface; EMD-6224). Atomic models for individual monomers are colored (ribbons; PDB: 3J9C). Membrane bilayer is shown as a cartoon for reference. **(b)** Conformational changes within a PA monomer upon pore formation. Residues in domain 2S that undergo the dramatic structural re-arrangement to form a β-barrel are indicated (orange). Domain 2C is highlighted by a blue circle. The pH-sensing loop 2β_10_–2β_11_ is colored green and blue for the prepore (PDB: 3KWV) [45] and pore (PDB: 3J9C) states, respectively. Inset shows a close-up of conformational changes within loop 2β_10_–2β_11_. Residue F427, in each conformation is shown as ball and stick model. **(c)** The φ-clamp is comprised of 7 phenylalanine residues (sidechain of F427 from each monomer shown as sticks) and controls translocation of pathogenic proteins into the cytosol by forming a 6 Å restriction in the pore lumen.

By implementing state-of-the-art methods in cryoEM, the structure of the PA pore revealed the toxin’s pH sensor and a mechanism for protein translocation in atomic detail. The PA pore is comprised of 4 individual domains arranged in a `flower-on-a-stem’ configuration (Figure 1a). During the prepore-to-pore transition, domain 2S undergoes dramatic structural rearrangements to form an extended β-hairpin that associates to form a long (105 Å) β-barrel pore (Figure 1a,b, orange ribbons). Low pH triggers the conformational change of a loop (2β_10_–2β_11_) (Figure 1b, inset) and the rotation of domain 2C (Figure 1b, blue circle), resulting in a ring of seven phenylalanine residues arranged within the lumen of the channel (Figure 1c). This seal, known as the φ-clamp, restricts passage of cations from the endosome to the cytosol during translocation of polypeptide chains [13]. The proton gradient generated across this transmembrane pore drives unidirectional transport, and the structure provides further evidence supporting a Brownian ratchet model for protein translocation [14].

## Aerolysin superfamily

The aerolysin superfamily comprises a group of β-PFPs characterized by a mushroom-like architecture, in which a central stem forms the β-barrel pore [15]. Similar to the anthrax PA toxin, soluble monomers bind the surface of membranes where they oligomerize and undergo a dramatic conformational change to form a transmembrane pore. For some members, such as lysenin, oligomerization is triggered by sphingomyelin-binding [16]. By contrast, aerolysin itself requires a proteolytic activation before oligomerization [17]. Recent high-resolution cryoEM structures of the lysenin pore [18^••^], together with a number of aerolysin pore intermediates [19^••^], have significantly advanced our understanding of how water-soluble monomers convert to transmembrane assemblies.

**Figure 2:**
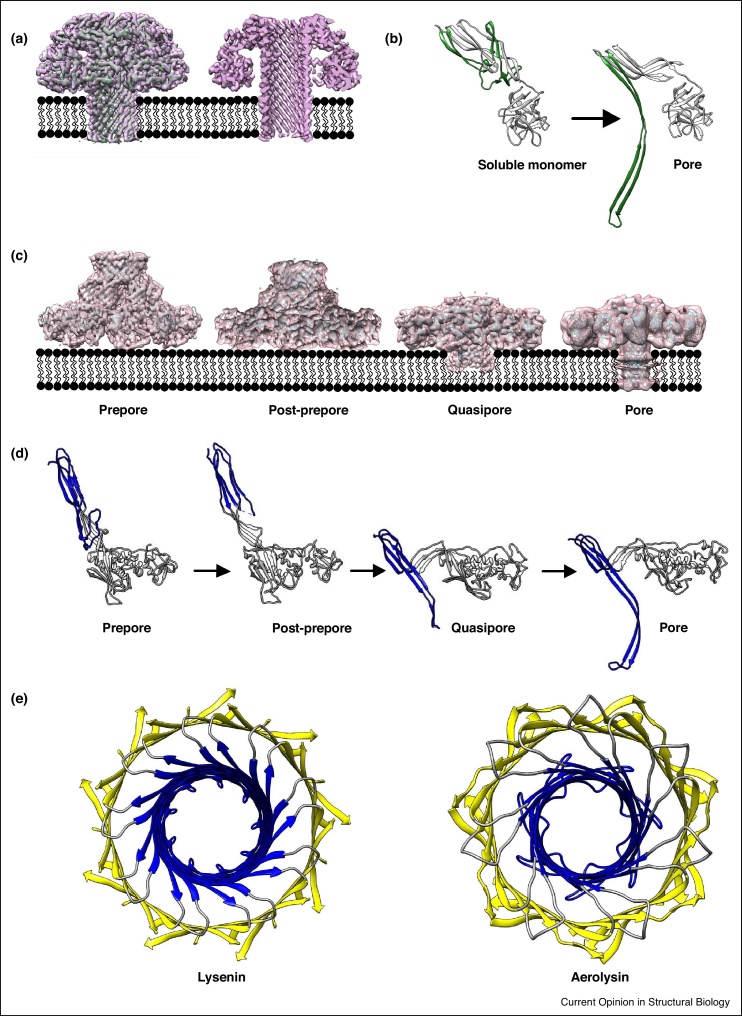
CryoEM structures of aerolysin-like β-PFPs. **(a)** CryoEM reconstruction of a mature lysenin pore at 2.9 Å resolution (magenta surface; EMD-8105) and corresponding atomic model (green ribbons; PDB: 5GAQ). Right panel is a cross-section of the structure. Membrane bilayer is shown as a cartoon for reference. **(b)** Conformational changes within a lysenin monomer upon pore formation. Residues that undergo the dramatic structural re-arrangement to form a β-barrel are indicated (green). Atomic models for monomers in soluble (PDB: 3ZXD) and membrane-inserted (PDB: 5GAQ) states are shown. **(c)** CryoEM structures (light pink surfaces) and corresponding atomic models (blue ribbons) of steps along the pathway to forming an aerolysin pore. Prepore at 3.9 Å resolution (EMD-8185; PDB: 5JZH), post-prepore at 4.5 Å resolution (EMD-8188; PDB: 5JZW), quasipore at 4.5 Å (EMD-8188; PDB: 5JZW) and final pore at 7.9 Å resolution (EMD-8187; PDB: 5JZT) states are shown from left to right. **(d)** Atomic model of an aerolysin monomer within the heptameric oligomer for each state. Residues that undergo the structural re-arrangement to form a β-barrel are highlighted as blue ribbons. **(e)** Atomic models for lysenin (left panel) and aerolysin (right panel) highlighting the concentric double β-barrel fold for this family of β-PFPs. The inner and outer barrels are blue and yellow, respectively.

The structure of the lysenin pore provided the first atomic resolution information of how aerolysin-like proteins insert into membranes [18^••^,^20^]. A previous crystal structure of the soluble monomer defined the domain architecture and sphingomyelin-binding residues of the toxin [21]; however, the active transmembrane form remained elusive. Detergent solubilized lysenin is highly unstable and subject to aggregation at concentrations necessary for structural studies. Whereas this challenge was overcome for PA using a thin layer of amorphous carbon [12^••^], lysenin pores were adsorbed to holey carbon grids overlayed with graphene oxide [18^••^]. Graphene oxide is a support nearly transparent to the electron beam and results in higher signal-to-noise ratios of images and consequently improved image alignment accuracies [22]. The structure revealed that lysenin forms a nonameric pore whose β-barrel spans the length of the complex (97 Å) (Figure 2a). Upon pore formation, a flexible coil within the cap and receptor-binding domains (residues: V157-R159) facilitates a rotation of the cap domain towards the lumen of the barrel and triggers the toxin’s dramatic vertical collapse (20 Å) towards the membrane. The pore is formed by the restructuring of 5 β-strands and a 3_10_ helix of the soluble monomer into an elongated β-hairpin whose transmembrane residues are comprised of a flexible insertion loop (Figure 2b, green ribbons). The lumen of the pore is rich in serine and threonine; the outer face is lined by hydrophobic amino acids. Similar to other transmembrane β-barrels, the lysenin pore contains two aromatic rings separated by the width of the bilayer. These residues, together with a histidine triad near the outer leaflet, lie at the interface between polar headgroups and hydrophobic acyl chains of the lipid and may stabilize the pore in the membrane.

Although the lysenin structure provided new insights into the final membrane-inserted state, structural characterization of different steps along the pathway is required for deriving a complete molecular mechanism. Aerolysin, the archetypal member of the superfamily, has been used to visualise prepore intermediates trapped by the introduction of cysteine bridges. Low-resolution cryoEM reconstructions of disulphide-locked aerolysin mutants revealed a swivelling of domains and vertical collapse of the complex during the prepore-to-pore conversion [23]. With images now recorded using a DED, these same mutants produced near-atomic resolution reconstructions (Figure 2c) [19^••^]. The improved signal-to-noise ratio of the DED-collected images enabled them to be sorted computationally and classified into homogeneous subsets. The structure of the aerolysin prepore revealed a heptameric oligomer whereby rotation of domain 4 drives the circular association of β-sandwich domains for each monomer. The prepore assembles into a novel concentric double β-barrel arrangement held together by a network of hydrophobic interactions (Figure 2e). During pore formation, the two barrels work together like a piston to rupture the bilayer. The inner β-barrel elongates and the protein undergoes a vertical collapse of nearly 40 Å towards the target membrane (Figure 2d). The piston injects hydrophobic loops at the tips of the β-hairpins into the membrane that then migrate laterally to anchor the complex like a rivet.

## Membrane attack complex perforin/cholesterol dependent cytolysin superfamily

**Figure 3:**
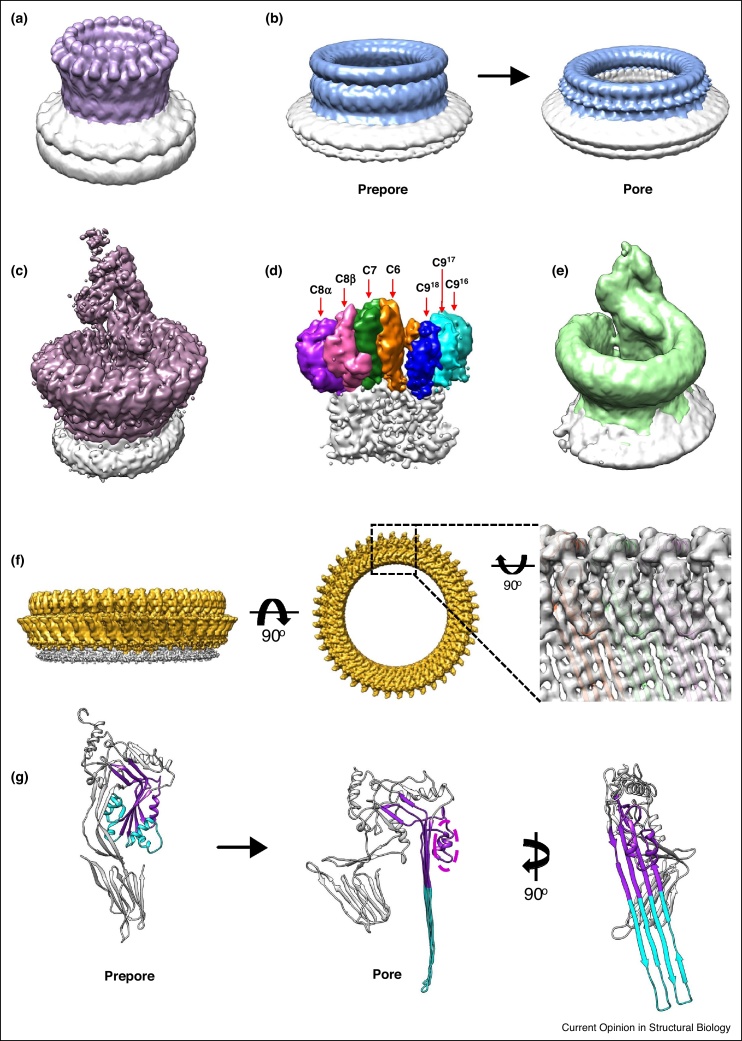
CryoEM structures of β-PFPs from the MACPF/CDC superfamily. (a,b) CryoEM structures of **(a)** perforin pore at 28.5 Å resolution (purple surface; EMD-1769) and **(b)** pneumolysin (PLY) prepore (28 Å; EMD-1106) and pore (28 Å resolution; EMD-1107) complexes (blue surface) before the ‘resolution revolution’. Density for the liposome membrane is grey. **(c)** Sub-nanometer (8.5 Å) cryoEM structure of the detergent solubilized MAC pore (purple surface; EMD-3134). Transmembrane region and detergent belt are grey. **(d)** Close-up of the MAC MACPF-rim colored according to component proteins (C6, C7, C8β, C8α, and C9 monomers); density corresponding to the β-barrel is grey. **(e)** Subtomogram average (28 Å resolution) of the MAC on liposomes (green surface; EMD-3289); density for the liposome membrane is in grey. **(f)** CryoEM reconstruction of the PLY pore at near-atomic (4.5 Å) resolution (EMD-4118). Protein density is gold; amphipol ‘belt’ is grey. Inset shows a close-up of interactions between PLY monomers (colored ribbons; PDB: 5LY6), as viewed from the inner face of the β-barrel. Density for the HTH motif and barrel β-strands is visible (grey surface). **(g)** Atomic models of PLY monomers in soluble (PDB: 5AOE) and transmembrane (PDB: 5LY6) conformations. The MACPF central β-sheet is colored purple. Residues that undergo the helix-to-hairpin transition during pore formation are light blue. The HTH motif is highlighted by a magenta dotted circle; the remainder of PLY is grey.

By contrast to the narrow diameter of PA and aerolysin pores (30 Å), members of the membrane attack complex perforin/cholesterol dependent cytolysin (MACPF/CDC) superfamily form giant β-barrel pores in target cell membranes. These complexes vary widely in stoichiometry (ranging 13–50 proteins) [24,25^••^,^26^] and can leave lesions up to 300 Å in the bilayer [27]. MACPF/CDC-containing proteins are one of the most prolific β-PFPs; they are secreted by bacteria as well as eukaryotic immune cells [28]. The MACPF/CDC fold is defined as a central kinked β-sheet with helical regions that unfurl into membrane-inserted β-hairpins upon pore formation. A number of low-resolution cryoEM structures of prepore and pore complexes on liposomes [26,27,29,30] (Figure 3a,b) illustrated how MACPF/CDC domains were oriented with respect to the membrane, however the molecular basis underlying these transitions remained unclear.

The membrane attack complex (MAC) is a MACPF-containing human immune pore that ruptures the cell membranes of pathogens. Contrary to β-PFPs solved-to-date, the MAC pore is not a symmetric ring [25^••^]. The complex is comprised of 22 proteins (7 unique polypeptide chains), making it a challenge to biochemically isolate intact assemblies and to obtain a homogenous population of particles for high-resolution cryoEM. A number of crystal structures of complement components [31,32], together with a low-resolution reconstruction of a soluble regulated MAC [31], provided some insight into the inactive forms of the complex; however, it was not until new developments in membrane protein biochemistry, coupled with technical advances in cryoEM, that the complete pore was solved at sub-nanometer resolution (Figure 3c,d). Detergents lower surface tension, making it challenging to achieve thin ice. A new class of detergents (neopentyl glycol) with two hydrophilic heads and two lipophilic tails were essential for increasing MAC stability while considerably reducing the percentage of detergent required in the freezing buffer. Similar to anthrax PA toxin, an amorphous carbon layer overlaid on the cryoEM grid was used to adsorb detergent solubilized MAC and prevent aggregation. Although classification of DED-collected images was implemented, limited particle numbers prevented extensive analysis of MAC heterogeneity. An electron cryo-tomography study of MACs in liposomes demonstrated a wide range of pore complex assemblies [33^•^]. The MAC structure provided clarity on the stoichiometry of component proteins and showed they were arranged in a non-canonical, split-washer architecture (Figure 3c–e). Furthermore, the structure revealed a novel asymmetric β-barrel pore in which some hairpins do not fully penetrate the lipid bilayer. By contrast to symmetric and homo-oligomeric β-PFPs solved to-date, this structure was the first to investigate how a break in symmetry could impact pore formation and raised new questions about how these complexes interact with their lipid environment.

Secreted by the pathogen *Streptococcus pneumoniae*, pneumolysin (PLY) is a CDC-containing β-PFP that binds cholesterol to form lytic pores on human cells. Previously, low resolution cryoEM structures of prepore and pores in model membranes showed a dramatic vertical collapse [27] (Figure 3b), reminiscent of the aerolysin pore transition (Figure 2). The atomic resolution cryoEM structure of a solubilized PLY pore (Figure 3f) has now identified specific amino acids that govern this conformational change [34^••^]. As with the MAC, stabilizing extracted pores was crucial for structural studies. Detergents heavily influenced the oligomeric state of PLY and introduced preferred orientations of the particle when adsorbed on the carbon-coated cryoEM grid. Exchanging detergent for amphipols led to the stabilization of homogenous PLY pores and resulted in a more complete angular coverage in the reconstruction. Amphipols are a new type of surfactant that stabilize membrane proteins in a detergent-free environment and enable better control over ice-thickness and protein distribution during sample vitrification [35]. The dominant 42-fold symmetric pores were further isolated *in silico* using 2D and 3D classification of DED-collected images. The resulting high-resolution reconstruction revealed electrostatic charge complementarity between monomers and inter-protein salt-bridges that stabilize the giant β-barrel. As observed for aerolysin and lysenin pore structures, large-scale rotations of PLY domains accompany a vertical collapse of the toxin towards the membrane. A helix–turn–helix motif within domain 3, HTH (Figure 3g, magenta dotted circle), unlatches the transmembrane residues, enabling their helix-to-hairpin transition (Figure 3g, cyan ribbons). Neighbouring β-hairpins associate to form a giant (260 Å) β-barrel whose lumen is highly polar. By contrast, the outer surface of the barrel is nearly all hydrophobic, with a ring of aromatic residues anchoring the pore within the membrane.

## Future perspectives and conclusions

**Figure 4:**
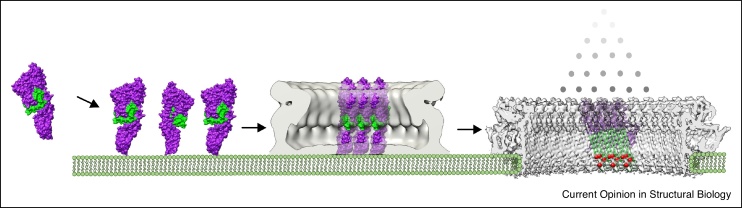
Structural re-arrangements underpinning β-PFP pore formation. Despite differences in size, sequence and oligomeric state, β-pore forming proteins share a general molecular mechanism for rupturing the lipid bilayer. Here, the major steps are highlighted using PLY as an example. Soluble monomers (surface-rendered, PDB: 5AOD) are secreted and bind target cell membranes (cartoon schematic). Monomers oligomerize (grey prepore map, EMDB-1106) on the bilayer. Subsequently, the protein undergoes dramatic structural re-arrangements, in which the transmembrane region (green) is remodelled to form a membrane-spanning β-hairpin. Cα carbons of aromatic residues within the transmembrane region are shown as red spheres.

Despite the diversity of β-PFPs, these structures reveal a number of conserved mechanisms of pore formation (Figure 4). Large-scale domain rotations accompany the aqueous-to-transmembrane transition. These conformational changes unlatch membrane-inserting residues, allowing them to restructure and pierce the bilayer. The final pore state is comprised of a β-barrel that extends beyond the width of the membrane. Two rings of aromatic residues lie at the interface between polar lipid headgroups and hydrophobic tails of the bilayer, thus anchoring the barrel’s position within the outer and inner leaflets. Electrostatic interactions play a key role in mediating both oligomerization interfaces as well as interactions with lipids.

β-PFPs are one of many biological systems that have benefitted from the ‘resolution revolution’ in cryoEM. Direct electron detectors have been the single biggest hardware advance and have greatly increased the signal to noise level in cryoEM images. Improved signal-to-noise ratios in the data have enabled a number of computational algorithms to correct for beam-induced specimen motion and to more accurately assign orientations to individual single-particles. In the past, difficulties in solving structures of oligomeric assemblies were due, in part, to challenges in symmetry determination. Low signal to noise ratios in cryoEM images contributed to weak peaks in rotational power spectrum used to assign point group symmetries. In addition, stoichiometric heterogeneity of the oligomer further complicated analysis of a bulk population. Mixtures unable to be purified biochemically can now be isolated *in silico* using 2D and 3D classification techniques. Indeed, extensive classification of cryoEM images for the recent Gasdermin A3 pore resolved subtle differences between oligomers comprised of either 27 or 28 subunits. The resulting high resolution structure (3.8 Å) enabled a complete atomic model to be built and showed how the pore binds the acidic lipid cardiolipin within the membrane [36^••^]. More sophisticated focused classification and refinement strategies involving density subtraction and creative masking approaches [37^••^] will likely result in a new wave of high-resolution β-PFP structures. Specifically, this strategy has been used to reveal structural heterogeneity and conformational variability within chemically identical subunits of the bacterial chaperone GroEL [38^••^]. Investigating the asymmetric nature of homo-oligomeric β-PFP assemblies may lead the uncovering of conformational hotspots that trigger pore formation.

Even with better detectors and new image processing algorithms, ultimately the sample and how it interacts with the EM grid during freezing remains a limiting factor for many β-PFPs. Grid supports have been instrumental in preventing aggregation of anthrax PA toxin, lysenin, MAC and PLY pore structures. However, supports can introduce further challenges by increasing background noise or preferred particle orientations. For lysenin, graphene oxide was used to minimize electron scattering from the support [18^••^]. Pre-treating amorphous carbon with polylysine improved the angular distribution of PA toxin on the grid [12^••^], while exchanging detergent for amphipols reduced over-represented top views for PLY pores. New computational tools that rapidly assess angular coverage and predict tilts to improve the distribution could be incorporated into data collection strategies [39,40^•^]. Tuneable grid supports are another area of future development that address many of the sample preparation challenges faced by β-PFPs. Hydrogenation of graphene through ionising with low-pressure gas can be an effective way to adjust protein adsorption [41]; however, it does not allow control of protein orientation. Self-assembling monolayers whose surface properties can be chemically modified have been used to improve the angular distribution of particles in ice [42]. Altering hydrophilicity of cryoEM grids through PEG-derived monolayers may offer an attractive alternative to detergent-treatment of supports, which may adversely impact membrane protein stability. Inkjet-dispensed vitrification methods [43,44], which allow small scale, reproducible sample preparation will substantially improve throughput for cryo-EM sample screening. As yields for purifying membrane proteins are notoriously low, this may provide a powerful high-throughput screening tool for optimizing β-PFP samples for cryoEM in the future.

## Conflict of interest statement

Nothing declared.

## References and recommended reading

Papers of particular interest, published within the period of review, have been highlighted as:

• of special interest
•• of outstanding interest
